# Photon-counting-detector CT outperforms state-of-the-art cone-beam and energy-integrated-detector CT in delineation of dental root canals

**DOI:** 10.1038/s41598-025-87081-w

**Published:** 2025-01-23

**Authors:** Stephan Rau, Martin Peter Pichotka, Alexander Rau, Marco Reisert, Markus Jörg Altenburger, Rainer Schmelzeisen, Fabian Cieplik, Fabian Bamberg, Maximilian Frederik Russe, Wiebke Semper-Hogg

**Affiliations:** 1 Department of Diagnostic and Interventional Radiology, Faculty of Medicine, Medical Center – University of Freiburg, University of Freiburg, Hugstetter Str. 55, 79106 Freiburg, Germany; 2 Division of Medical Physics, Department of Diagnostic and Interventional Radiology, Faculty of Medicine, University Medical Center Freiburg, University of Freiburg, Freiburg, Germany; 3 Department of Neuroradiology, Faculty of Medicine, Medical Center – University of Freiburg, University of Freiburg, Freiburg, Germany; 4 Department of Operative Dentistry and Periodontology, Faculty of Medicine, Medical Center – University of Freiburg, University of Freiburg, Freiburg, Germany; 5 Department of Orthodontics, Faculty of Medicine, Medical Center – University of Freiburg, University of Freiburg, 79106 Freiburg, Germany; 6 Department of Oral and Maxillofacial Surgery, Faculty of Medicine, Medical Center – University of Freiburg, University of Freiburg, Freiburg, Germany

**Keywords:** Tomography, X-ray computed, Photon-counting CT, Photon-counting detectors, CBCT, Oral anatomy, Dental pulp

## Abstract

This experimental phantom study investigates current standard of care protocols in cone beam computed tomography (CBCT), energy-integrating-detector (EID) CT, and photon-counting-detector (PCD) CT regarding their potential in delineation of dental root canals. Artificial accessory canals (diameters: 1000, 600, 400, 300 and 200 μm) were drilled into three bovine teeth mounted on a bovine rib as a jaw substitute. The phantom was scanned in two dental CBCTs, two EID-CTs and a PCD-CT using standard clinical protocols. Scans from a micro-CT served as reference standard. Spatial resolution was evaluated via line profiles through the canals, whereby visibility compared to surrounding noise and compared to the ground truth were assessed. PCD-CT was able to delineate all artificial canals down to 200 μm diameter. In CBCT and EID-CT canals could only be reliably detected down to 300 μm. Also, PCD-CT showed a considerably smaller width-divergence from the ground trough with 4.4% at 1000 μm and 35.1% at 300 μm compared to CBCT (13.5 and 72.9%) and EID-CT (10.1 and 115.7%). PCD-CT provided superior resolution, accurate size measurement, and enhanced detection of small dental root canals, thereby offering improvements in diagnostic capabilities compared to CBCT and EID-CT systems.

## Introduction

Dental root canal treatment is a common endodontic procedure aimed at preserving teeth with infected or inflamed pulp^1^. The root canal system is of complex morphology with main and accessory canals^2^. Thus, successful therapy relies on accurate visualization of the root canal system. Untreated parts of the canal system can lead to persistent symptoms and treatment failure^2,3^. The diameter of the main root canal measures down to 330–370 μm^4,5^, whereas calcified root canals or accessory canals can be considerably smaller^6^. Therefore, high-resolution imaging of dental structures is essential for precise treatment planning and improved patient outcomes.

Highest resolution in computed tomography (CT) can be achieved in micro-CT systems as they provide an excellent spatial resolution of 5–50 μm. However, apart from the limited scan size, micro-CT requires high radiation doses which are unsuitable for in vivo imaging in humans^7,8^.

Cone beam computed tomography (CBCT) is widely established in dental imaging due to its lower radiation dose and higher spatial resolution compared to conventional energy-integrated detector (EID)-CT^9^. However, the image quality of CBCT can be impaired by artifacts and noise, limiting diagnostic accuracy^10^. Although CBCT allows for reconstructions with voxel sizes of down to 75 μm^11,12^, the spatial resolution is considerably lower, depending on factors like physical pixel size of the sensor, gray-level resolution, reconstruction techniques, and motion artifacts. To this date, evidence of in vitro spatial resolution is still inconsistent ranging from 100 to 500 μm^13,14^.

EID-CT has also been used in dental imaging, offering faster acquisitions with lower sensitivity to motion artifacts compared to CBCT^15^. However, the spatial resolution of EID-CT is generally lower than that of CBCT limited to the detector specifications with a resolution of down to 400 μm^16^.

Recently, clinically available photon-counting detector (PCD)-CT has emerged as a promising technology that offers improved spatial resolution as well as reduced radiation dose compared to EID-CT^17,18^. The energy-resolving detectors enable higher spatial resolution of down to 150 μm and less electronic noise^16,19^.

Initial experiences with PCD-CT in dental and bone imaging yielded promising results^17,20–24^, though no systematic analysis of the spatial resolution in comparison to the established CBCT has been conducted. Therefore, the aim of this exploratory study was to compare the performance of clinical standard protocols in CBCT, EID-CT, and PCD-CT scanners in dental imaging using a dental phantom with artificial accessory canals with standardized diameters simulating dental accessory root canals.

## Materials and methods

### Phantom preparation

While human teeth are difficult to obtain due to regulatory and ethical reasons, bovine teeth and bovine bone material were selected to simulate clinical conditions, as bovine teeth have comparable anatomical and morphological characteristics^25^. Also, available human teeth have a greater anatomical diversity^26^, whereas the morphological diversity of the bovine teeth is low due to the similar animal age of the available material, thereby ensuring a more standardized phantom setup. Especially in endodontics, bovine ribs have been used in various studies on accuracy evaluation of CBCT to simulate the jaw line, as they are of similar anatomical characteristics, too, e.g. in length determination of root canals^11,27,28^.

Therefore, bovine material from dead cows from the local slaughterhouse was used. The local veterinary authority has approved the usage of the tissues for research purpose (City of Freiburg im Breisgau; Office for Public Order - Veterinary Department AZ: 32.602.01, June 19th, 2024).

In line with the exploratory design of this study, a small sample size of three teeth was used to evaluate the initial feasibility and performance of the scanning systems in delineating artificial dental root canals. Therefore, three bovine mandibular incisors were used for the study as they have a similar anatomy to human teeth. The extracted teeth were cleaned and embedded in kneadable silicone for proper positioning in the drilling device. The labial root surfaces were flattened using a histological grinding device (Struers GmbH, Willich, Germany) to ensure perpendicular drilling into the dentine, preventing drill failure. Holes were drilled along the tooth axis to standardize positioning and ensure reproducibility relative to the root canal utilizing a bench drill (Bosch PBD 40, Gerlingen-Schillerhöhe, Germany).

Accordingly, the three teeth were prepared by drilling five artificial accessory canals of different diameters per tooth (1000 μm, 600 μm, 400 μm, 300 μm, and 200 μm) using a high-precision micro drill system simulating a human-like dental phantom. Monitoring of the accuracy of the drill diameter was performed with a Mitutoyo micrometer with 1 μm resolution (Mitutoyo Europe GmbH, Neuss, Germany). Subsequently, the teeth were inserted in a bovine rib to simulate a jaw while the orientation of the teeth ensured comprehensive support within the rib bone. The phantom was then stored in a humid environment to prevent dehydration and maintain the integrity of the prepared teeth.

### Imaging systems

The dental phantom was scanned using different CT systems employing standard clinical protocols. An overview is displayed in (Table 1). For CBCTs, the phantom was positioned with orientation of the synthetic mandible in plane to the scan direction and for the EID- and PCD-CTs with orientation orthogonal to the scan direction to resemble clinical routine scanning.

**Table 1:** Overview of acquisition and reconstruction parameters.

Scanner type	Model		Tube voltage (kVp)	Exposure (mAs)	Voxel size (µm)	Other parameters
PCD-CT	NAEOTOM alpha (siemens)		120	35	200	CARE kv IQ Level 110, pitch 0.85
EID-CT1	SOMATOM Definition edge plus (siemens)		120	27–71	500	Collimation 0.6 mm; pitch 0.8
EID-CT2	SOMATOM force (siemens)		130	31–91	400	Collimaiton 0.6 mm; pitch 0.85
CBCT1	Planmeca viso G7 (Planmeca, Helsinki, Finland)	Large FOV: 100 × 60 mm	100	32	150	Tube current 9.0 mA; Scan time 3.56 s
		Small FOV: 30 × 60 mm	100	32	150	Tube current 9.0 mA; Scan time 3.56 s
CBCT2	3D Accuitomo 170 (Morita, Osaka, Japan)	Large FOV: 140 × 100 mm	89	87	250	Tube current 4.9 mA; Scan time 17.5 s
		Small FOV: 40 × 40 mm	89	87	80	Tube current 4.9 mA; Scan time 17.5 s
Micro-CT	SkyScan 1276 (Bruker)		100	n/a	10	Exposure 2007 ms; rotation steps 0.25° (360° rotation)

*PCD-CT* photon-counting CT, *EID-CT1* energy-integrating detector CT1, *EID-CT2* energy-integrating detector CT2, *CBCT1* cone beam CT 1, *CBCT2* cone beam CT 2, *FOV* field of view.

#### Dental CBCT

Two commercially available dental CBCT scanners were used. The field of view was selected to completely visualize the artificial jaw, simulating radiographs within the context of clinical routine. As an additional clinical reference, the CBCT scans were repeated deploying dedicated single tooth scans modes for endodontic imaging with smallest possible voxel sizes. The scans were performed with the following parameters:

CBCT1 (Planmeca Viso G7, Planmeca, Helsinki, Finland): tube voltage 100 kVp, tube current 9.0 mA with 32.4 mAs. Free adjustment of FOV: Large field of view with 100 × 60 mm and 150 μm voxel size and for the small field of view 30 × 60 mm with 150 μm voxel size. The scan time was 3.56 s.

CBCT2 (3D Accuitomo 170, Morita, Osaka, Japan): tube voltage 89 kVp, tube current 4.9 mA with 87 mAs. Predefined FOV sizes: Large field of view with 140 × 100 mm and 250 μm voxel size and for the small field of view 40 × 40 mm with 80 μm voxel size. The scan time was 17.5 s.

#### EID-CT

Two clinical third-generation EID-CT scanners (EID-CT1: SOMATOM Definition Edge Plus, Siemens Healthineers, Forchheim, Germany and EID-CT2: SOMATOM Force, Siemens Healthineers, Forchheim, Germany) were utilized. The scans were repeated three times in different gantry positions to account for image noise, employing an ultra-high-resolution protocol. For EID-CT1: tube voltage 130 kVp, 27–71 mAs, collimation 0.6 mm, pitch 0.8, and reconstructed voxel size 500 μm. For the EID-CT2: tube voltage 120 kVp, 31–94 mAs, collimation 0.6 mm, pitch 0.85, and reconstructed voxel size 400 μm.

#### Clinical PCD-CT

A first-generation clinical PCD-CT scanner (NAEOTOM Alpha, Siemens Healthineers, Forchheim, Germany) was employed. Again, the scan was repeated three times in different gantry positions to account for image noise, using the following parameters: tube voltage 120 kVp, CARE kv IQ Level 110, tube current 35 mAs, pitch 0.85, and reconstructed voxel size of 200 μm.

#### Micro-CT

A micro-CT scanner (Bruker SkyScan 1276, Kontich, Belgium) served as the reference standard with an acceleration voltage 100 kVp, camera type XIMEA MH110XC-KK-TP with a pixel size of 17.468 μm, and image pixel size of 10.28 μm. The scan was performed for each tooth individually using a beam-filter combination of Al 0.5 and Cu 0.03 mm and an exposure time of 2007 ms per step with rotation steps of 0.25°. A complete 360° rotation was performed during the acquisition to ensure comprehensive coverage and high-resolution imaging.

### Image reconstruction and analysis

All acquired scans were reconstructed using dedicated software provided by the respective manufacturers. For CBCT, a filtered back-projection algorithm with a Feldkamp-Davis-Kress kernel was used with a voxel size of 0.15 mm for CBCT1 and 0.25 mm for CBCT2. Micro-CT images were reconstructed by Bruker’s NRecon software (Bruker SkyScan 1276, Kontich, Belgium), using a symmetrical boxcar filter with a filter strength of 8, and a beam hardening correction strength of 51%.The EID-CT1 scans were reconstructed in axial series using a standard bone kernel (Hr68) with smallest possible parameters of 0.5 mm slice thickness and 0.3 mm increment and Advanced Modeled Iterative Reconstruction with strength 3. The EID-CT2 scans were reconstructed in axial series using a standard high-definition bone kernel (Ur81) with smallest possible parameters of 0.4 mm slice thickness and 0.2 mm increment and Advanced Modeled Iterative Reconstruction with strength 3. The PCD-CT scan was reconstructed in axial series using a standard high-definition bone kernel (Hr80) with smallest possible parameters of 0.2 mm slice thickness and 0.1 mm increment and Quantum Iterative Reconstruction strength 3.

**Fig. 1:**
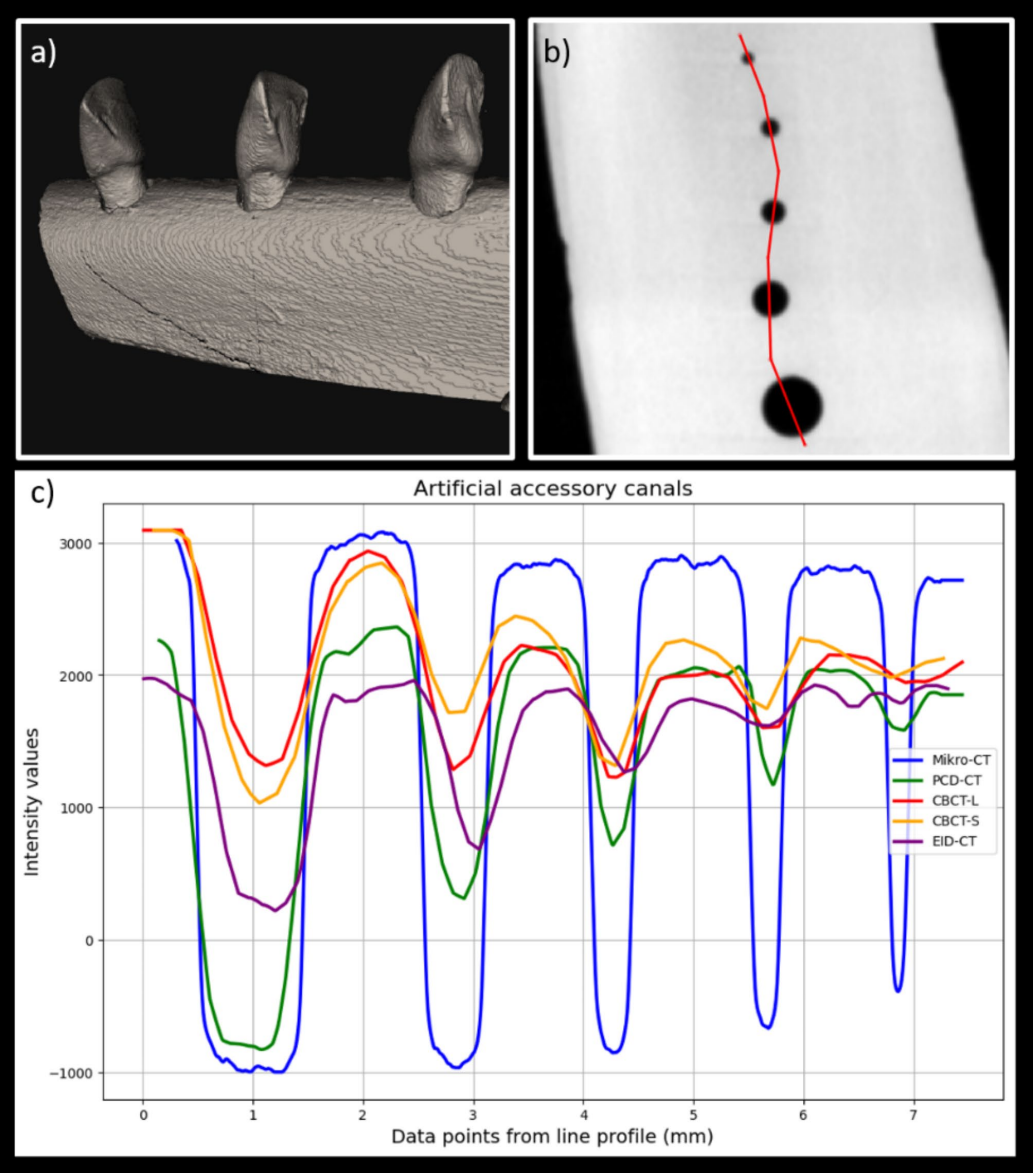
Phantom model with examples of line profiles. (**a**) Volume rendering of a CT scan of bovine teeth in the rib mount. (**b**) Axial reconstruction from the micro-CT with visualization of the artificial accessory canals and depiction of the line profile (red line). (**c**) Graphical representation of the intensity values (corresponding to the non-calibrated Hounsfield units (HU) values) of the line profiles through a tooth in micro-CT (blue), photon counting-detector (PCD) CT (green), energy-integrated-detector (EID) CT (violet) using EID-CT2 and cone-beam (CB) CT (red) using CBCT1 with the large (CBCT-L) and small (CBCT-S) FOV. *PCD-CT* photon-counting CT, *EID-CT* third-generation energy-integrating detector CT, *CBCT* cone beam CT, *HU* hounsfield units.

The reconstructed images were exported in DICOM format for further analysis. An in-house custom-built software tool for image post-processing (The Nora Medical Imaging Platform Project, Freiburg, Germany^29^) was used to extract the image crops of the artificial accessory canals from each tooth individually. Here, the image segments with the artificial holes were extracted for each tooth individually as a separate DICOM data set for the further analysis. Secondly, a line profile was placed orthogonal to the artificial accessory canals directions through the center of each artificial accessory canal using multiplanar reconstructions as depicted in (Fig. 1). In Fig. 1, the line profiles illustrate the contrast between the different scanning systems, whereby only a subset is displayed to provide better clarity and visualization. The detailed analyses of all scanners are presented in the corresponding tables. The visibility of the artificial accessory canals was assessed by comparing the signal intensity of an artificial accessory canal in the line profile to the surrounding noise. The noise was omitted via region of interest (ROI) measurements next to the canal in the surrounding dental matter (standardized size of 0.016 cm²). An artificial accessory canal was considered visible if the signal within the accessory canal was equal or two times larger than the standard deviation of the noise. The width of an artificial accessory canals was measured in all scanners based on the area of low signal intensity of the artificial accessory canal in the line profile. Here, the edges of the canal were defined as the full width at half density-minimum of the line profile compared to the baseline density of the tooth which was determined via the ROI measurements. Subsequently, the width was than compared to the ground truth values obtained from the micro-CT scans to obtain the relative error.

### Image quality

General image homogeneity was evaluated by calculating the coefficient of variation (CV) via ROI measurements next to the artificial accessory canals in each scan for each method using the standard deviation (*σ*) and the mean value (*μ*):

$$\:CV=\frac{{\sigma\:}_{ROI}}{{\mu\:}_{ROI}}\:$$

### Radiation dose

The radiation dose was measured using the Computed Tomography Dose Index (CTDI) and the dose-length product (DLP) for all CT scans and the dose-area product (DAP) for the CBCT scans.

### Statistical analysis

Descriptive statistics were used to summarize the results, including the width for each artificial accessory canal in each imaging system. The accuracy of the size measurements was evaluated by calculating the relative error (%) compared to the ground truth. The detection rates of the artificial accessory canals were reported as percentages of identified artificial accessory canals for each imaging system per artificial accessory canal size. All values for the artificial accessory canal detection and width for all scanners were reported as mean values of each tooth in all available scans. The values for image quality and radiation dose were compared via ANOVA with a Tukey post-hoc test. The alpha-level was set to 0.05. All statistical analyses were performed using Python Software (version 3.10.12).

## Results

### Artificial accessory canal visibility

**Fig. 2:**
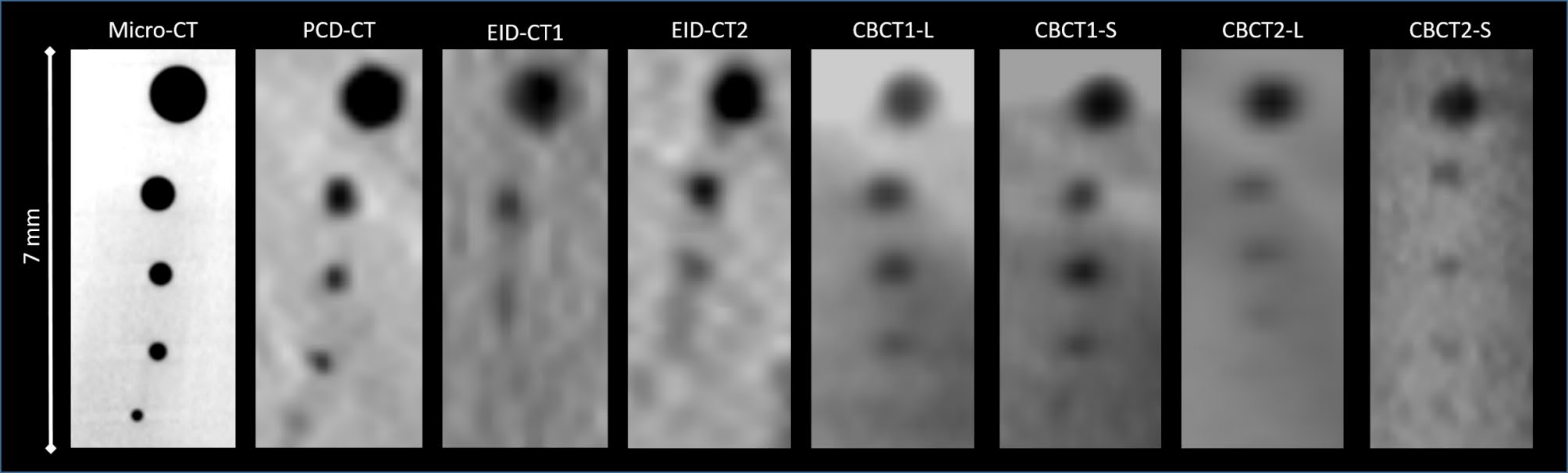
Exemplary images of the artificial accessory canals in all scanners. Analogous slices tangentially through the direction of the artificial accessory canals in the same tooth in all tested imaging systems (L for large FOV and S for small FOV). For visualization of the size, the white line on the right represents a distance of 7 mm. *PCD-CT* photon-counting CT, *EID-CT* third-generation energy-integrating detector CT, *CBCT* cone beam CT, *HU* hounsfield units.

The PCD-CT system demonstrated superior performance in detecting the artificial accessory canals compared to the other clinical imaging systems (Table 2). PCD-CT was the only one modality capable of reliably delineating all artificial accessory canals in each of the three teeth down to 200 μm diameter, corresponding to a 100% detection rate. In contrast, the CBCT1 could reliably delineate all artificial accessory canals down to 300 μm and both EID-CTs failed to detect artificial accessory canals with 200 and 300 μm, with varying success rates. These statistical findings can be confirmed visually. A head-to-head comparison of the image reconstructions between the scanners orthogonal to the trajectory of the artificial accessory canals in a single tooth is provided in (Fig. 2). Compared to the other clinical scanners using the protocol for clinical routine PCD-CT provides the best delineation in particular of the small artificial accessory canals. Noteworthy, using the dedicated endodontic scan mode in CBCT1 and CBCT2 lead to higher detection rates than clinical standard CBCT protocols with one missed artificial accessory canal with 200 μm in both scanners but did not outperform the detection in PCD-CT with a larger field of view.

**Table 2:** Artificial accessory canal detection rates.

		1000 μm (%)	600 μm (%)	400 μm (%)	300 μm (%)	200 μm (%)
PCD-CT		100	100	100	100	100
EID-CT1		100	100	78	44	22
EID-CT2		100	100	100	89	11
CBCT1	Large FOV: 100 × 60 mm	100	100	100	100	0
	Small FOV: 30 × 60 mm	100	100	100	100	89
CBCT2	Large FOV: 140 × 100 mm	100	100	33	0	0
	Small FOV: 40 × 40 mm	100	100	100	100	89

*PCD-CT* photon-counting CT, *EID-CT1* energy-integrating detector CT1, *EID-CT2* energy-integrating detector CT2, *CBCT1* cone beam CT 1, *CBCT2* cone beam CT 2, *FOV* field of view.

### Artificial accessory canal width measurements

The micro-CT, serving as the reference standard, clearly visualized all artificial accessory canal down to 200 μm in all three teeth with 0.48–2.9% difference to the drill head size. Upon comparison of width measurements from the artificial accessory canals obtained in the clinical systems with the micro-CT, the PCD-CT provided the most accurate results. PCD-CT showed a considerably smaller divergence from the true artificial accessory canals width, with a relative error of 4.4% at 1000 μm and 35.1% at 300 μm. In comparison, CBCT1 had a relative error of 13.5% at 1000 μm and 72.9% at 300 μm, while the EID-CT2 showed a relative error of 13.5% at 1000 μm and 115.7% at 300 μm. Detailed results with standard deviations are presented in (Table 3).

**Table 3:** Relative errors of the artificial accessory canal width compared to micro-CT measurements.

Rel. error		1000 μm (%)	600 μm (%)	400 μm (%)	300 μm (%)	200 μm (%)
PCD-CT		4.4 ± 3.7	9.2 ± 3.9	13.4 ± 12.5	35.1 ± 21.4	85.6 ± 53.4
EID-CT1		15.5 ± 4.9	26.7 ± 18.2	88.7 ± 25.8	122.5 ± 70.3	135.8 ± 30.7
EID-CT2		10.1 ± 4.1	14.7 ± 13.5	67.0 ± 34.1	115.7 ± 67.2	156.7 ± 0
CBCT1	Large FOV: 100 × 60 mm	13.5 ± 4.2	10.5 ± 6.0	23.7 ± 17.0	72.9 ± 34.5	n/a
	Small FOV: 30 × 60 mm	9.2 ± 3.4	4.1 ± 4.2	27.5 ± 7.3	70.5 ± 7.4	392.4 ± 63.9
CBCT2	Large FOV: 140 × 100 mm	23.8 ± 7.6	39.1 ± 10.7	41.1 ± 0	n/a	n/a
	Small FOV: 40 × 40 mm	13.4 ± 4.4	6.2 ± 1.0	14.2 ± 2.1	42.4 ± 21.4	194.8 ± 13.4

*PCD-CT* photon-counting CT, *EID-CT1* energy-integrating detector CT 1, *EID-CT2* energy-integrating detector CT 2, *CBCT1* cone beam CT 1, *CBCT2* cone beam CT 2, FOV field of view.

Although the dedicated endodontic scan mode in CBCT1 and CBCT2 provided an improved detectability, a higher errors in the width determination compared to the PCD-CT was observed. Particularly for the smallest artificial accessory canal with 200 μm, the relative error of 392.4 ± 63.9 and 194.8 ± 13.4% for CBCT1 and CBCT2 was substantially higher than for PCD-CT with 85.5 ± 53.4%.

### Image quality

Quality assessment of the reconstructed images revealed notable differences in image quality among the imaging systems. PCD-CT provided equivalent CV to both CBCTs in both field of view settings, whereas both EID-CT systems (EID-CT1 and EID-CT2) provided significantly higher CV compared to PCD-CT. Micro-CT images served as the reference standard with CV of 0.015 ± 0.001, which was significantly lower than all CT imaging systems, whereas CBCT2 and the small field of view in CBCT1 performed equally. A comprehensive overview of the CV values and the comparison of CV between scanners is provided in (Tables 4 and 5).

**Table 4:** Coefficient of variation for all scanners and scan modes.

Scanner	Mean value	Standard deviation
Micro-CT	0.0151	0.0035
PCD-CT	0.0402	0.0167
EID-CT1	0.0586	0.0169
EID-CT2	0.0702	0.0263
CBCT1-L	0.0565	0.0386
CBCT1-S	0.0255	0.0055
CBCT2-L	0.0326	0.008
CBCT2-S	0.0350	0.0130

*PCD-CT* photon-counting CT, *EID-CT1* energy-integrating detector CT 1, *EID-CT2* energy-integrating detector CT 2, *CBCT1-L* cone beam CT 1 large field of view, *CBCT1-S* cone beam CT 1 small field of view, *CBCT2-L* cone beam CT 2 large field of view, *CBCT2-S* cone beam CT 2 small field of view.

**Table 5:** Matrix for Tukey post-hoc-test for the coefficient of variation across scanners.

		Mikro-CT	PCD-CT	EID-CT1	EID-CT2	CBCT1-L	CBCT1-S	CBCT2-L	CBCT2-S
Mikro-CT	Mean difference	–	−0.0251	−0.0435	−0.0551	−0.0414	−0.0103	−0.0174	−0.0198
	p-value	–	< 0.001	< 0.001	< 0.001	< 0.001	0.846	0.248	0.120
PCD-CT	Mean difference		–	−0.0183	−0.0300	−0.0163	0.0148	0.0077	0.005
	p-value		–	< 0.001	< 0.001	0.116	< 0.001	0.391	0.987
EID-CT1	Mean difference			–	−0.0116	0.0021	0.0331	0.0260	0.024
	p-value			–	0.109	1.000	< 0.001	< 0.001	0.002
EID-CT2	Mean difference				–	−0.0137	−0.0448	−0.0377	−0.0353
	p-value				–	0.293	< 0.001	< 0.001	< 0.001
CBCT1-L	Mean difference					–	0.0310	0.0239	0.0216
	p-value					–	< 0.001	0.025	0.066
CBCT1-S	Mean difference						–	−0.0071	−0.0095
	p-value						–	0.977	0.896
CBCT2-L	Mean difference							–	−0.0024
	p-value							–	1.000
CBCT2-S	Mean difference								–
	p-value								–

*PCD-CT* photon-counting CT, *EID-CT1* energy-integrating detector CT 1, *EID-CT2* energy-integrating detector CT 2, *CBCT1-L* cone beam CT 1 large field of view, *CBCT1-S* cone beam CT 1 small field of view, *CBCT2-L* cone beam CT 2 large field of view, *CBCT2-S* cone beam CT 2 small field of view.

### Radiation dose

For the PCD-CT, the radiation dose was substantially lower compared to the EID-CTs with a mean CTDI of 6.01 mGy and a DLP of 52.43 mGy*cm against a mean CTDI of 9.29 mGy and a DLP of 85.83 mGy*cm for the EID-CT1 and with a mean CTDI of 8.79 mGy and a DLP of 78.57 mGy*cm for the EID-CT2. The DAP was 207 mGy/cm² for CBCT1 and 2140 mGy/cm² for CBCT2 and for the single tooth scans 590 mGy/cm² for CBCT1 and 402 mGy/cm² for CBCT2.

## Discussion

The results of this study indicate a superior performance of PCD-CT in dental imaging compared to state-of-the-art CBCT and EID-CT systems. PCD-CT was able to reliably detect and accurately measure the dimensions of artificial accessory canals simulating dental root canals down to 200 μm in diameter, outperforming both CBCT and EID-CT.

The superior performance of PCD-CT can be attributed to its innovative detector technology, which directly converts X-ray photons into electrical signals without collimation, enabling higher spatial resolution, improved contrast-to-noise ratio, and reduced radiation dose compared to EID-CT systems^16,30^. PCD-CT is therefore suited for the visualization of fine anatomical details, such as narrow root canals and accessory canals. This is particularly important in endodontics, in which the detection and treatment of these structures are crucial for the success of root canal therapy potentially improving patient outcomes^2,3^. So far, CBCT has been widely used in dental imaging due to its higher spatial resolution and lower radiation dose compared to conventional EID-CT^31,32^. However, the results of our study suggest that the spatial resolution of examinations with voxel sizes down to 150 μm and regular field of view in modern CBCT may be limited in detecting and accurately measuring small structures like accessory canals, especially canals with a diameter smaller than 300 μm. This finding is consistent with a previous study on CBCT, reporting a spatial resolution to be around 500 μm^14^. Nonetheless, scans with a smaller field of view are recommended for the dedicated evaluation of accessory canals in endodontic imaging^33^. Here, the spatial resolutions was higher but the systems still came short in the delineation of the artificial accessory canals with 200 μm compared to PCD-CT. EID-CT, despite its widespread use in medical imaging, demonstrated insufficient performance among the artificial accessory canals with 300 μm and 200 μm. Although the newer EID-CT2 system outperformed the EID-CT1, it still fell short of the performance of PCD-CT. The higher noise levels and lower spatial resolution of EID-CT hampered its ability to detect and measure small artificial accessory canals accurately. The limited spatial resolution of EID-CT can be attributed to the detector specifications consisting of a scintillator with septa and a photodiode array with a cell-width of down to 250–630 µm^34^. This observation is in line with previous studies that reported the limitations of EID-CT in visualizing fine dental structures and the need for higher radiation doses compared to CBCT^31,35^. The image quality assessment using the coefficient of variation (CV) revealed that PCD-CT provided equivalent image homogeneity to CBCT systems, while both EID-CT systems had significantly higher CV values. This finding suggests that PCD-CT can maintain high spatial resolution without compromising image quality, which is essential for accurate diagnosis and treatment planning.

Beyond endodontics, PCD-CT has potential applications in other dental specialties. In implant dentistry, PCD-CT could provide more accurate measurements of alveolar bone dimensions and density, aiding in the selection of appropriate implant sizes and locations. In periodontics, PCD-CT may facilitate the early detection of periodontal bone loss and the assessment of periodontal regeneration therapies as it was already shown for CBCT, but with the advantage of an effective reduction of metal artifacts^36–38^. As a result of the improved capabilities of PCD-CT in dental imaging, opportunistic assessment of the jaws is feasible in scans of the facial region, for example in the context of examinations of the neck, paranasal sinuses or cervical spine. Hereby, high-resolution information about the dental anatomy can be obtained. Consequently, unnecessary repeated radiation exposure for patients might be avoided.

The limitations of this study include the use of a simplified phantom model with a limited sample size, which may not fully represent the complexity of human dental anatomy and the presence of surrounding soft tissues. However, the peripheral ends of the artificial canals were in direct contact with the surrounding bone imitating physiological conditions, which should minimize the impact of the missing soft tissue on the imaging results. Nonetheless, the use of a phantom allowed for a systematic comparison of the imaging systems’ performance in detecting and measuring small structures. Nevertheless, further research using larger sample sizes with extracted teeth or in vivo studies should validate our findings. Additionally, the study focused on the visualization and measurement of artificial accessory canals, which may not entirely reflect all challenges encountered in clinical endodontic imaging, such as the presence of calcifications, root fillings, and metal artifacts. Of note, single tooth scans in CBCT with smaller voxel sizes offered higher detectability while still being inferior to PCD-CT in terms of detectability and in the precision of the width of the artificial accessory canals. In addition, single tooth scan modes in CBCT represent a highly focused imaging technique, excluding substantial portions of the dental and facial system from the field of view. Also, the scan protocols for the EID-CTs and the PCD-CT were transferred from clinical routine with highest possible settings for image reconstructions, whereby the spatial resolution is influenced by the reconstruction parameters. The comparison of the radiation dose of CBCT to other CT systems is highly complex due to the different radiation geometry and requires complex physical calculations. Therefore, in the setting of this phantom study no further comparison was made.

In conclusion, PCD-CT demonstrated superior spatial resolution, more accurate size measurements, and enhanced detection of small structures simulating dental root canals compared to CBCT and EID-CT systems. This suggests that PCD-CT has the potential to improve diagnostic capabilities and treatment planning in dental imaging. The clinical implementation of PCD-CT in dental practice could lead to more precise endodontic treatments and improved patient outcomes by enabling the detection and management of accessory root canals that might otherwise be missed with conventional imaging techniques.

## Data Availability

The datasets generated during and/or analysed during the current study are available from the corresponding author on reasonable request.

## Abbreviations

CT: Computed tomography
PCD-CT: Photon-counting CT
EID-CT: Energy-integrating detector CT
CBCT: Cone beam CT
QIR: Quantum iterative reconstruction
ROI: Region-of-interest
CTDI: CT dose index
DLP: Dose-length product
DAP: Dose-area product
